# Validation of combined Monte Carlo and photokinetic simulations for the outcome correlation analysis of benzoporphyrin derivative-mediated photodynamic therapy on mice

**DOI:** 10.1117/1.JBO.24.3.035006

**Published:** 2019-03-14

**Authors:** Karl W. Beeson, Evgueni Parilov, Mary Potasek, Michele M. Kim, Timothy C. Zhu

**Affiliations:** a Simphotek, Inc., Newark, New Jersey, United States; b University of Pennsylvania, Department of Radiation Oncology, Philadelphia, Pennsylvania, United States

**Keywords:** medical device, photodynamic therapy, solid cancer therapy

## Abstract

We compare previously reported benzoporphyrin derivative (BPD)-mediated photodynamic therapy (PDT) results for reactive singlet oxygen concentration (also called singlet oxygen dose) on mice with simulations using a computational device, Dosie™, that calculates light transport and photokinetics for PDT in near real-time. The two sets of results are consistent and validate the use of the device in PDT treatment planning to predict BPD-mediated PDT outcomes in mice animal studies based on singlet oxygen dose, which showed a much better correlation with the cure index than the conventional light dose.

## 1 Introduction

Photodynamic therapy (PDT) has been approved by the U.S. Food and Drug Administration for the treatment of esophageal cancer, non-small cell lung cancer, Barrett’s esophagus, and age-related macular degeneration.^1^^–^^6^ Two types of PDT killing mechanisms are known to exist when utilizing light-activated photosensitizers (PSs) to generate reactive oxygen species that kill cancer cells. Type I PDT generates superoxide anion (${O_{2}}^{-}\cdot$) and other secondary oxygen species, such as hydroxyl radicals (HO·) or hydrogen peroxide ($H_{2}O_{2}$) for treatment.^7^ Many commonly used PSs are type II. Type II PDT is a complex process^7^ involving the interactions of the local photosensitizer concentration [PS], the local light fluence rate (intensity or ϕ), and the local oxygen concentration [O32] in order to produce singlet oxygen (O12). Furthermore, the local light fluence rate depends on light transmission through intervening tissue having local absorption coefficient, $\mu_{a}$, and local reduced scattering coefficient, $\mu_{s}^{\prime }$. The complexity of the type II PDT process has made monitoring of treatments difficult and unreliable. Many clinical studies have not resulted in optimal treatment due to the lack of reliable dosimetry for singlet oxygen.^6^

Several types of dosimetry have been utilized to measure the response of cancer to PDT, including incident light dose^8^ (or incident fluence, which equals incident fluence rate times the treatment time), PDT dose^9^ (the time integral of PS concentration times the fluence rate), and singlet oxygen dose^10^^–^^12^ (also denoted as reacted singlet oxygen concentration or [O12]rx). The most common PDT dosimetry method used by most PDT researchers and medical practitioners is the relatively simple procedure of measuring incident in-air fluence rather than using a more comprehensive method that accounts for patient-specific *in-vivo* fluence distribution, which depends on tissue optical properties, photochemical parameters, treatment time, molecular oxygen concentration, etc. The drawback to the simplicity of incident fluence dosimetry is that treatment results for the same light dose can vary considerably for different patients.

PDT dose is a better alternative to light dose as it accounts not only to tissue light distribution but also to treatment/patient-specific unevenness of [PS]. Although O12 is thought to be the major cause of cell toxicity for type II PSs, O12 is very difficult to directly measure. However, the dosimetry quantity [O12]rx can be calculated by combining photokinetics (PK) equations with calculations for light transport.^13^ This dosimetry quantity can then be used to predict treatment outcomes.

Modeling the photophysics/photochemistry of PDT is very challenging and a significant problem for improving the PDT treatments. Over the past several years, Zhu et al.,^9^^–^^13^ Foster et al.,^14^^–^^17^ Wilson et al.,^18^^,^^19^ and other groups^20^^–^^23^ have proposed or described various PDT models to compute limited aspects of PDT, such as light transport, variation of optical parameters, and aspects of oxygen concentration and flow. In addition, over the past several years, there have been developments of treatment planning and monitoring tools using the laser light dose as the main criterion^24^^–^^28^ of PDT effectiveness but without the critical photophysics. For example, Davidson et al.^29^ developed a treatment planning software package and employed it in a phase II clinical trial of Tookad™-mediated PDT of persistent prostate carcinoma following radiation therapy. Treatment plans were based on pretreatment MRI images. Optical properties were determined by fitting a diffusion-based model to the *in vivo* fluence rate measurements. The treatment plan was evaluated by the light dose distribution superimposed on the MRI images of the largest volume. Light distribution calculations were verified by comparing fluence rate measurements made prior to Tookad™ infusion, with fluence rate data extracted during treatment. Treatment results were measured six months post-treatment with biopsies. In patients where the light dose was less than $23{\;J/\mathrm{cm}}^{2}$, none had a cancer-free biopsy response. In tumors treated with light dose greater than $23\;{J/\mathrm{cm}}^{2}$, 62% had cancer-free biopsies after six months. The dosimetry concentrated on the light optical properties of the tissue and the light fluence delivered to various regions of the prostate. Results were not conclusive based solely on light dosimetry. A phase I clinical trial of motexafin lutetium-mediated PDT of prostate cancer was performed at Penn.^30^^–^^32^ An integrated dosimetry and treatment planning system was developed at Penn utilizing a kernel-based algorithm to calculate light fluence rate distribution in a heterogeneous medium.^33^^,^^34^ The integrated system included a PDT dosimetry system to determine the 3-D distribution of tissue optical properties or diffuse optical tomography,^35^^,^^36^ as well as the distribution of PS concentration and oxygen saturation (${\mathrm{StO}}_{2}$).^37^ It utilized an optimization algorithm to determine the optimal positions of sources based on either light fluence^38^ or singlet oxygen.^39^ Preliminary results show that motexafin lutetium-mediated PDT will have an effect on the prostate-specific antigen (PSA) level when the light dose is larger than $150{\;J/\mathrm{cm}}^{2}$.^32^ PDT treatment planning has also been done using FullMonte tetrahedral-based MC software that may be more accurate if tissue surfaces are curved.^40^ Since the geometry for this study is planar, tetrahedral-based MC simulations are not expected to modify the results.

Commercial treatment planning and monitoring software, “Interactive Dosimetry by Sequential Evaluation” (iDOSE^®^), was developed by Spectracure, Inc. It provided light dose plans with optical fiber positions based on three dimensional (3-D) tissue models generated from ultrasound.^26^ The software optimizes the fiber positions and provides a clinically acceptable plan for laser light in the tumor. A first monitoring sequence is performed after the optical fibers are in place. Initially, homogeneous optical properties are assumed for each cluster of optical fibers and initial monitoring is performed. At specific intervals, the light is interrupted and a monitoring evaluation test is performed. Tissue optical properties are obtained using the same fibers used for delivering the therapeutic light. This enables one to determine the effective attenuations and update the light dose. In a phase I/II clinical study, the Spectracure system was used with mTHPC (Foscan^®^) in the treatment of patients with histologically proven untreated, organ-confined prostate cancer. Initially, a conservative light dose of $5\;{J/\mathrm{cm}}^{2}$ was used to limit damage to surrounding tissue. Following treatment, the PSA level was higher than expected for complete treatment and it was concluded that the light dose of $5\;{J/\mathrm{cm}}^{2}$ was insufficient. In a later preclinical study, in male canines, this group suggested that the threshold light dose should be in the range of 20 to $30\;{J/\mathrm{cm}}^{2}$ for effective PDT with mTHPC in the treatment of prostate cancer. However, iDOSE^®^ does not include the PK of the PS and oxygen, which makes the treatment outcome dependent on a sufficient level and evenness of PS and oxygen concentrations.

These studies^9^^–^^39^ demonstrate the need for a more predictive, comprehensive, fast, and complete computer simulation for PDT. In this study, we expand on simulations developed at the University of Pennsylvania, Department of Radiation Oncology (Penn).^9^^–^^13^ The aim is to create a more complete simulation of PDT treatment that can be used for animal studies as well as in the clinic in near real-time. Simphotek, Inc. has developed Dosie™, an integrated proprietary computational device consisting of proprietary software and designated hardware. In the study presented here, we compare fluence rate and [O12]rx results using Dosie™ to experimental and approximate simulations for superficial BPD-mediated PDT obtained by Kim et al.^10^ at Penn in animal studies. The Dosie™ device can also be used to simulate any PS for which the PK parameters are known. In the future, we plan to validate Dosie™ in clinical trials.

## 2 Simulation Software and Designated Hardware

### 2.1 Integrated Computational Device

The Dosie™ hardware and proprietary software incorporates, in one integrated computational device, Monte Carlo (MC) simulations of light transport, light fluence rate, and light dose (fluence) in target regions as well as PK simulations of singlet oxygen dose ([O12]rx) and PDT dose. The MC approach is ideal for simulating light transport in biological tissue.^41^^,^^42^ This method has been greatly expanded over the years and has been successfully ported^43^ onto graphical processor units (GPU) to improve its performance. The subsequent PK simulations use the MC results and PS properties as inputs to calculate the resulting PDT dose and [O12]rx.

**Fig. 1:**
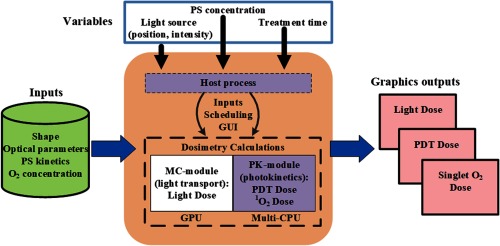
Schematic diagram for Dosie™ integrated computer and software computational device. The computer includes both a GPU and a multicore CPU. The software first performs a MC simulation of light transport that is followed by a PK simulation.

A schematic diagram of Dosie™ is shown in Fig. 1. The host process is an independent component of the software that is responsible for scheduling all PDT-related numerical calculations. It provides access to and controls two modules: light transport (MC-module) and PK-module. A graphical user interface (GUI) enables users to define PDT session simulation parameters, to launch and control simulations, and to visualize the output. The parameters needed for the numerical calculations are added from a database or from direct optical measurements before treatment. They include the 3-D target shape (prepared by third-party software, generated by measurements, such as CT or MRI), the optical parameters of the target (light scattering and light absorption coefficients), PK parameters for the PS (see Table 1 for BPD parameters), and the initial oxygen concentration. The variable parameters include laser power and location(s), PS (drug) concentration, and treatment time. The first calculation step is to use the MC-module to determine the light intensity (fluence rate) and light dose (fluence) at every microscopic point in the tumor. CUDA calculations are performed on a massively parallel GPU processor to simulate the paths of tens of millions of light rays. The calculated light intensity field is transferred from GPU memory to the host memory to launch the PK-module on the CPU processers to calculate the PDT PK that includes the light-PS-excitation (and photobleaching), the PS-oxygen excitation to generate singlet oxygen, and the singlet oxygen reaction with the target (including singlet-oxygen induced photobleaching). Comprehensive proprietary graphics, developed by Simphotek in the Dosie™ device, are used to visualize 2-D and 3-D outputs of the light fluence (light dose) and fluence rate, PDT dose, and singlet oxygen dose as well as concentrations of PS, ground-state oxygen, and cancer killing the [O12]rx at every microscopic point in the 3-D target for the duration of the treatment. This enables the researcher or physician to localize areas of possible undertreatment or overtreatment in the operating room while the patient is undergoing treatment and make corrections.

**Table 1:** BPD simulation parameters.^10^^,^^44^

Simulation parameters for BPD^®^
$g(\mu M\;s^{-1})=1.7$
δ(μM)=33
β(μM)=11.9
$\sigma ({\mu M}^{-1})=1.8\times 10^{-5}$
$\xi ({\mathrm{cm}}^{2}\;{\mathrm{mW}}^{-1}\;s^{-1})=0.055$

### 2.2 MC-Module (Light Transport)

The MC-module is based on MC photon transport that allows modeling light propagating in a heterogeneous translucent medium. Such a medium is characterized by high likelihood of (mostly forward) light scattering events occurring within short distances (submillimeter scale) and by a high albedo (absorption is typically two orders of magnitude smaller than the scattering). The MC-module numerically estimates the resulting 3-D maps of light fluence rates and light doses (fluences) within the PDT target translucent region (target volume) during a planned PDT session for a given arrangement of the incident or interstitially applied light sources.

For BPD simulation, a collimated light source is located in air and directed perpendicular to the surface of the target volume. Our modified proprietary version of MC models’ light propagation within voxel-based geometry, where the target volume is subdivided into voxels (cubes of identical sizes) so that the graphical output 3-D map of Dosie™ assigns one fluence rate value for each voxel. The target volume consists of heterogeneous media with known optical properties (i.e., absorption coefficient, scattering coefficient, scattering anisotropy factor, and refractive index) so that each voxel can be assigned a unique set of properties.

During MC simulations, millions of photon packets are launched toward the target volume, according to the light excitation distribution. Each packet—we will be using word “photon” instead for the rest of this section—has an initial weight of 1. Specular reflection/refraction is taken into account at the air-target surface interface by applying the Fresnel model for unpolarized light with the refractive index 1.4. Each photon can undergo multiple scattering events in the target volume until the photon either escapes the volume or is absorbed within the volume when its weight drops below a specified threshold. Within the medium, a photon propagates free, voxel by voxel, till the next scattering event. The length of such free propagation is randomly determined according to the scattering coefficient $\mu_{s}$. If the optical properties change while the photon travels from one voxel to another, the free path is properly adjusted. At each scattering event, a trajectory direction is determined by applying the Henyey–Greenstein phase function. The photon weight is gradually reduced while traveling from one voxel to another by a factor $e^{-\mu_{a}l}$, where l is the portion of its trajectory within each such a voxel. All fractions of the weight lost that occurred during propagation of each photon are deposited at every voxel it passes so that by the end of the simulation, we can estimate the fluence from the accumulated weights.

A voxel-based implementation of MC is relatively straightforward, as it does not require adding potentially time-consuming ray-boundary intersection tests. However, for geometries with multiple curved surfaces and/or for less scattering media, a boundary-based MC implementation may result in more accurate solutions due to more precise reflection/refraction calculations along the boundaries (e.g., as in this tetrahedral-based^40^ MC implementation).

MC simulations can be very time consuming (i.e., hours) for translucent media. The main challenge is to make the simulations run in near real-time (i.e., a minute or less). At the current state of the computer hardware, only parallel calculations are capable of reaching near real-time performance. MC-proprietary module software in Dosie™ is a GPU CUDA code (an extension to C++ programming language that allows scheduling identical compute kernels to run as threads on GPU Core processors in parallel; CUDA compute capability 5.0 has been used in Dosie™) with a performance target of completing target light transport simulations within minutes. Each CUDA thread performs a simulation of a photon propagation accessing the voxels’ optical properties of each encountered voxel from the device constant memory and depositing the weight’s losses to the device shared memory. The final fluence calculations are done in the host process after the accumulated weights are transferred from the device memory to the host memory using the proprietary Dosie™ device, including experimental hardware configuration of Core i7-6820HQ (16G memory, 64-bit) CPU and NVIDIA Quadro M2000M (640 cores, 4G memory, CUDA 5.0) GPU, the near-real time target performance has been achieved.

### 2.3 PK-Module

The PK-module of Dosie™ is a complicated and essential part of modeling PDT. Unlike the light transport, this is a quasi-quantum mechanical process in which the laser light energy (photon) is transformed into reactive chemical agents that kill cancer cells. The PK calculations are done for each voxel independent from other voxels. This allows the calculations to run in parallel by taking advantage of a multicore CPU architecture. PK Eqs. (1)–(3) are used to calculate the time evolution of the (ground state) PS concentration, $\left[S_{0}\right]$, the ground state oxygen concentration, [O32], and the reacted singlet oxygen concentration, [O12]rx, where the value $\left[S_{0}\right]$ is the BPD concentration and ϕ is the fluence rate. This is an initial value problem with the given initial fields $\left[S_{0}\right]$ (x,y,z,t=0)≥0 and [O32] (x,y,z,t=0)>0 while [O12]rx (x,y,z,t=0)=0, and with satisfying a non-negativity requirement at all times: $\left[S_{0}\right]$(t>0)≥0 and [O32] (t>0)≥0. The initial $\left[S_{0}\right]$ for each mice group is listed in Table 2. A variant of an embedded Runge–Kutta formulation RK5(4), adjusted to satisfy a non-negativity condition on a solution, is implemented in PK-module as a numerical proprietary C++ code to solve the system Eqs. (1)–(3). Previously, Penn solved^10^^,^^13^ a similar system using MATLAB internal solver. Simphotek’s PK-module has been validated against MATLAB calculations^45^ resulting in a perfect match for the published parameters. The equations are as follows:^10^^,^^13^

d[S0]dt+(ξσϕ([S0]+δ)[O23][O23]+β)[S0]=0, (1)

d[O23]dt+(ξϕ[S0][O23]+β)[O23]−g(1−[O23][O23](t=0))=0, (2)

d[O21]rxdt−(ξϕ[S0][O23][O23]+β)=0. (3)

**Table 2:** Summary of the results for all mice groups. Penn data are taken from Ref. 10. The optical power (column 6) used for the Dosie™ simulations equals the area of the 10-mm diameter emitting disc ($0.7854\;{\mathrm{cm}}^{2}$) times the Penn in-air fluence rate (column 2). The [O12]rx simulated results for 1.00-, 0.50- and 0.25-mm voxels are shown in columns 8, 9, and 10, respectively. The values in parentheses for columns 8, 9, and 10 are the mismatch to the Penn values (column 7).

1 Penn	2 Penn	3 Penn	4 Penn	5 Penn	6 Dosie™	7 Penn	8 Dosie™	9 Dosie™	10 Dosie™	11 Penn	12 Penn
Group	Incident in-air fluence rate ($\mathrm{mW}/{\mathrm{cm}}^{2}$)	Time (s)	Incident in-air fluence ($J/{\mathrm{cm}}^{2}$)	$[\mathrm{BPD}{]}_{\mathrm{pre}}$ (μM)	Power in 10 mm disc (W)	At 3 mm depth [O12]rx (mM)	[O12]rx (mM) 1.00 mm grid (% diff.)	[O12]rx (mM) 0.50 mm grid (% diff.)	[O12]rx (mM) 0.25 mm grid (% diff.)	K (1/days)	Index, CI
1	50	600	30	0.53	0.0393	0.39	0.4067 (+4.29)	0.4057 (+4.04)	0.4054 (+3.95)	0.40	0.0377
2	75	400	30	0.72	0.0589	0.45	0.4607 (+2.39)	0.4633 (+2.95)	0.4638 (+3.06)	0.38	0.0556
3	150	200	30	0.56	0.1178	0.29	0.2913 (+0.46)	0.2936 (+1.24)	0.2941 (+1.43)	0.40	0.0237
4	50	1400	70	0.73	0.0393	0.90	0.9147 (+1.63)	0.9200 (+2.23)	0.9213 (+2.36)	0.28	0.3151
5	75	1333	100	0.41	0.0589	0.60	0.5977 (−0.39)	0.6032 (+0.53)	0.6044 (+0.74)	0.37	0.1037
6	50	2700	135	0.50	0.0393	0.78	0.7839 (+0.50)	0.7910 (+1.41)	0.7928 (+1.64)	0.34	0.1646
7	75	1800	135	0.53	0.0589	0.82	0.8246 (+0.56)	0.8319 (+1.45)	0.8337 (+1.67)	0.32	0.2139
8	150	900	135	0.58	0.1178	0.85	0.8626 (+1.48)	0.8684 (+2.17)	0.8698 (+2.33)	0.28	0.3240
9	75	2000	150	0.84	0.0589	1.30	1.3094 (+0.72)	1.3196 (+1.51)	1.3222 (+1.71)	0	1
10	100	1500	150	0.66	0.0785	1.03	1.0292 (−0.07)	1.0373 (+0.70)	1.0393 (+0.90)	0.11	0.7432
11	75	3333	250	0.58	0.0589	0.96	0.9600 (+0.00)	0.9630 (+0.31)	0.9637 (+0.38)	0.25	0.3878
12	150	1667	250	0.77	0.1178	1.26	1.2599 (−0.01)	1.2651 (+0.40)	1.2664 (+0.50)	0	1
13	150	2000	300	0.77	0.1178	1.27	1.2728 (+0.22)	1.2758 (+0.46)	1.2766 (+0.52)	0	1
14	150	2333	350	0.81	0.1178	1.35	1.3432 (−0.50)	1.3450 (−0.37)	1.3454 (−0.34)	0	1
15	0	0	0							0.41	0

In these equations, g is the oxygen supply rate to the tissue, δ is the low-concentration correction, β is the oxygen quenching threshold concentration, σ is the specific photobleaching ratio, and ξ is the macroscopic maximum oxygen supply rate. The parameter values specific for the PS, BPD, are given in Table 1. The initial oxygen concentration, [O23] (x,y,z,t=0), is assumed to be 40  μM for all simulations.

### 2.4 Graphical User Interface

**Fig. 2:**
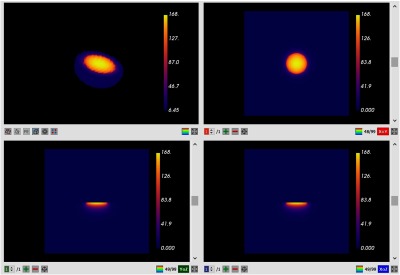
Dosie™ GUI screenshots: MC fluence rate results using 0.50-mm voxels for an incident fluence rate of $50\;{\mathrm{mW}/\mathrm{cm}}^{2}$. Volume rendering is done to visualize 3-D fluence rate map in the upper left view, whereas other views show three 2-D cross-sections (slices) of the map—sagittal, coronal, and transverse views.

The calculations are broken into individual sessions. Using the GUI created in Dosie™, users can create sessions, archive current sessions, browse and search for previous sessions, define PDT simulation parameters, and launch simulations. The visualization component in Dosie™ includes tools to render 2-D and 3-D simulation and geometry data. It enables users to visualize and analyze dose maps resulting from MC and PK calculations (see Fig. 2) to visually inspect under- and overdosed regions. Visualization view has a control panel that lists all dose maps currently available for visualization for each completed session or allows uploading maps from the file system and creating groups of maps for intersession analysis.

The GUI shows four conventional views for visualization of the target data: one model view with a 3-D map and three 2-D slice views of the individual 2-D slices resulting in a 2-D sagittal (side) view, a 2-D coronal (front) view, and a 2-D transverse (horizontal) view. The slicing planes can be moved to visualize different cross-sections of the 3-D calculations shown in model view. This enables the user to investigate the fine, near microscopic details of the treatment in the target.

In this study, we will focus on the MC fluence rate and [O12]rx singlet oxygen dose—the quantity that showed a strong correlation with PDT outcome in recent animal studies.^10^ The voxel-based MC simulations of light transport run over 100×100×100 grids of either ${\left(1\;\mathrm{mm}\right)}^{3}$, ${\left(0.5\;\mathrm{mm}\right)}^{3}$, or ${\left(0.25\;\mathrm{mm}\right)}^{3}$ voxels. From the resulting fluence rate maps, the subsequent PK calculations determine the singlet oxygen dose or [O12]rx for each voxel. We show that Dosie™ and the Penn preliminary calculations give nearly equivalent results.

## 3 Experimental and Computational Methods

### 3.1 Experimental Procedures Summary

Superficial PDT was done at Penn using mice having radiation-induced fibrosarcoma (RIF) tumors. The cancerous mice were injected with the PS benzoporphyrin derivative (BPD, Visudyne^®^) followed by PDT treatments.^10^ The authors define a cure index for the mice and analyze the correlation of the cure index to the three types of doses: light fluence dose, PDT-dose, and singlet oxygen dose. Penn denotes the singlet oxygen dose as the reacted singlet oxygen concentration, or [O12]rx.

Penn researchers divided the mice into 15 groups, each containing 3 to 5 mice. PDT was delivered to 14 groups. The 15th group was the control group that did not receive either BPD or PDT. RIF cells were cultured and 30  μl (at $1\times 10{}^{7}\text{ cells}/\mathrm{ml}$) were injected in the right shoulders of 6- to 8-week old female C3H mice. The resulting tumors were treated with PDT when the tumors were ∼3 to 5 mm in diameter and ∼3-mm thick. The mice were injected with BPD 3 h before treatment. The mice were then treated with a 10-mm diameter, 690 nm, collimated light beam from an 8 W diode laser (B&W Tek Inc., Newark, Delaware). The incident in-air fluence was 30 to $350\;{J/\mathrm{cm}}^{2}$, which corresponded from 50 to $150\;{\mathrm{mW}/\mathrm{cm}}^{2}$ fluence rate.

Simulations of singlet oxygen dose require knowledge of the BPD concentration in the tumor, the optical properties of the tumor, and the initial ground-state oxygen concentration, [O32], in the tumor. Penn did fluorescence measurements on each mouse using a custom multifiber spectroscopic contact probe before treatment to determine the initial BPD concentration.^46^ The average initial BPD concentrations, ${\left[\mathrm{BPD}\right]}_{\mathrm{pre}}$, for each group of mice, are listed in Table 2. Tissue optical properties for the tumors, $\mu_{a}$ and $\mu_{s}^{\prime }$, were also measured using a multifiber contact probe.^44^ For the simulations, it is assumed that the tissue has a single set of optical properties, $\mu_{a}=0.69\;{\mathrm{cm}}^{-1}$ and $\mu_{s}^{\prime }=11\;{\mathrm{cm}}^{-1}$, which are typical values for tumors in all mice groups.

To determine a cure index, CI, Penn measured tumor dimensions daily for 14 days after PDT and calculated tumor volumes using the formula:^47^ $V=\pi a^{2}b/6$, where a and b are diameters of the width and length axes. A tumor regrowth rate for each tumor was determined by the best fit to an exponential of the form, $e^{kt}$, where t is the time (in units of days) after PDT. Then CI is obtained as follows:^10^

$$\mathrm{CI}=1-\frac{k}{k_{\mathrm{ctr}}},$$ (4)

where k is the tumor regrowth rate and $k_{\mathrm{ctr}}$ is the regrowth rate for the control group that was not injected with BPD and did not undergo light illumination. Penn results for CI are listed in Table 2.

### 3.2 Calculations of Fluence Rate and [O12]rx

The bulk of Penn simulations have been performed in MATLAB. To determine fluence rate versus tissue depth at the center of the light beam, Penn used the previously determined, one-dimensional (1-D) analytical expression to approximate MC results at the center of a 10-mm diameter treatment light beam:^48^

$$\frac{\phi }{\phi_{\text{air}}}=(1-be^{-\lambda_{1}d})(C_{2}e^{-\lambda_{2}d}+C_{3}e^{-\lambda_{3}d}),$$ (5)

where the parameters $\lambda_{1}$, $\lambda_{2}$, $\lambda_{3}$, b, $C_{2}$, and $C_{3}$ for a 10-mm diameter beam are functions^48^ of $\mu_{a}$, $\mu_{s}^{\prime }$, $u_{\mathrm{eff}}$, and $R_{d}$ ($R_{d}$ is the diffuse reflectance at the interface between air and tissue). For $\mu_{a}=0.69\;{\mathrm{cm}}^{-1}$ and $\mu_{s}^{\prime }=11\;{\mathrm{cm}}^{-1}$, then $R_{d}=0.321$.

Full 3-D MC simulations of fluence rate versus depth were done for the same mice data using MC-module of Dosie™. The 3-D simulations of Dosie™ allow the visualization of the results in all three dimensions rather than just the 1-D results obtained by Penn only at the center of the treatment beam. Each MC simulation launched 20 million collimated light rays directed perpendicular to the target surface from a 10-mm-diameter disc (the area is $0.7854\;{\mathrm{cm}}^{2}$).

## 4 Results

### 4.1 Fluence Rate Calculations

**Fig. 3:**
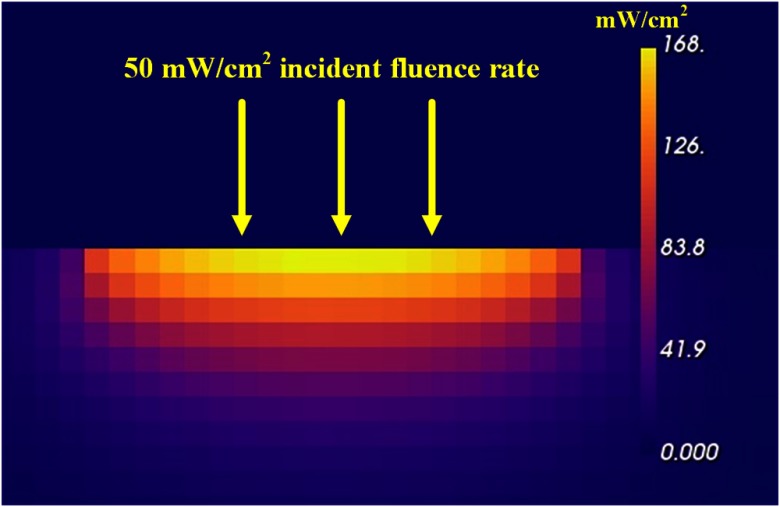
YZ slice of Dosie™’s MC fluence rate results using 0.50-mm voxels through the center of the target volume for an incident fluence rate of $50\;{\mathrm{mW}/\mathrm{cm}}^{2}$.

In Figs. 2 and 3, we show Dosie™’s partial screenshots of MC light transport results for the fluence rates inside the target volume using 0.50 mm voxels, ${\left(0.50\;\mathrm{mm}\right)}^{3}$ cubes, an incident fluence rate of $50\;\mathrm{mW}/{\mathrm{cm}}^{2}$ assumes that the tissue has a single set of optical properties, $\mu_{a}=0.69\;{\mathrm{cm}}^{-1}$ and $\mu_{s}^{\prime }=11\;{\mathrm{cm}}^{-1}$. The Dosie™ MC runtime was 21 s for 20 million photons.

There are four views in the MC output graphics in Fig. 2. In the upper left, a portion of the target volume with significant fluence values is rendered. The view is interactive allowing the user to rotate and magnify the target volume. In this example, the volume is tilted to view, where the light is incident on the target surface. The other three views are an XY slice (upper right), an YZ slice (lower left), and an XZ slice (lower right). The positions of each slice can be moved by the user within the target volume. Each slice can be expanded to fill the graphical image space. The graphics showing the expanded YZ slice (with added labels) of fluence rate values is shown in Fig. 3.

Note that although the incident fluence rate is $50\;{\mathrm{mW}/\mathrm{cm}}^{2}$, the peak fluence rate induced just below the surface is $168\;{\mathrm{mW}/\mathrm{cm}}^{2}$ (as shown on the scale at the right), which is 3.36 times larger than the incident fluence rate. This enhanced fluence rate occurring just under the surface of the target volume is typical for scattering media, where light can be scattered in all directions and can undergo total internal reflection under the target surface, which adds significantly to the magnitude of the primary incident light.

**Fig. 4:**
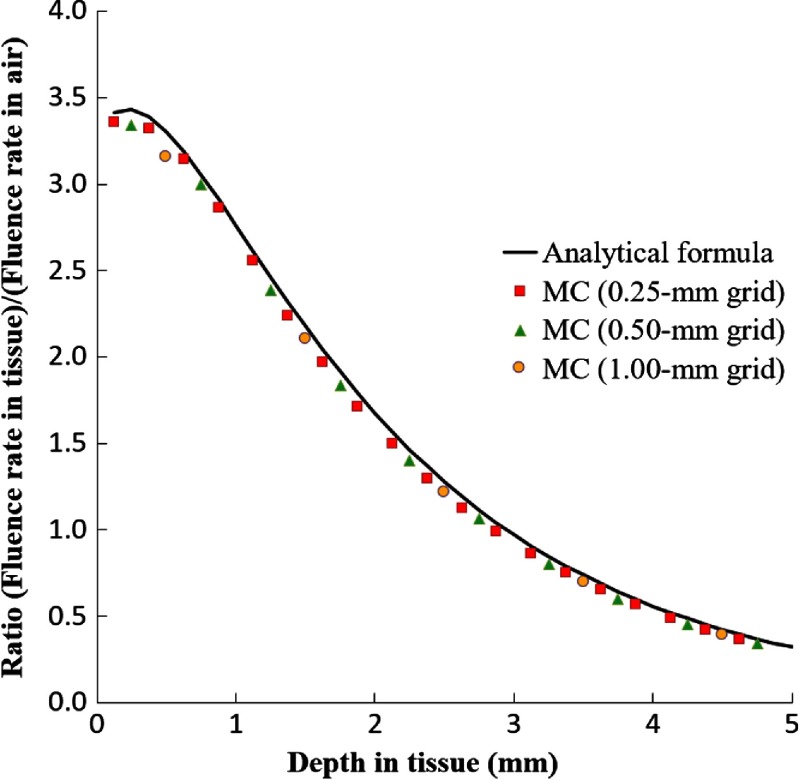
The ratio (fluence rate in tissue)/(fluence rate in air) versus depth in tissue that compares an analytical fit^48^ to Dosie™’s MC results.

A comparison of the analytical formula, Eq. (5), to MC calculations of Dosie™ for the ratio (fluence rate in tissue)/(incident fluence rate in air) versus depth in tissue is shown in Fig. 4. For MC, the results at the center of the beam using 1.0, 0.5, and 0.25 mm grid steps are done for an incident fluence rate of $50\;{\mathrm{mW}/\mathrm{cm}}^{2}$. For the 1.0-mm grid, the first voxel (centered at 0.5 mm depth) misses the initial higher ratio of fluences at ∼0.25  mm depth in the target. The 0.5-mm and 0.25-mm grids more accurately resolve the fluence ratios for the smaller depths. Consequently, the MC results for such grids are approximately identical to the analytical approximation.

### 4.2 PK Calculations

**Fig. 5:**
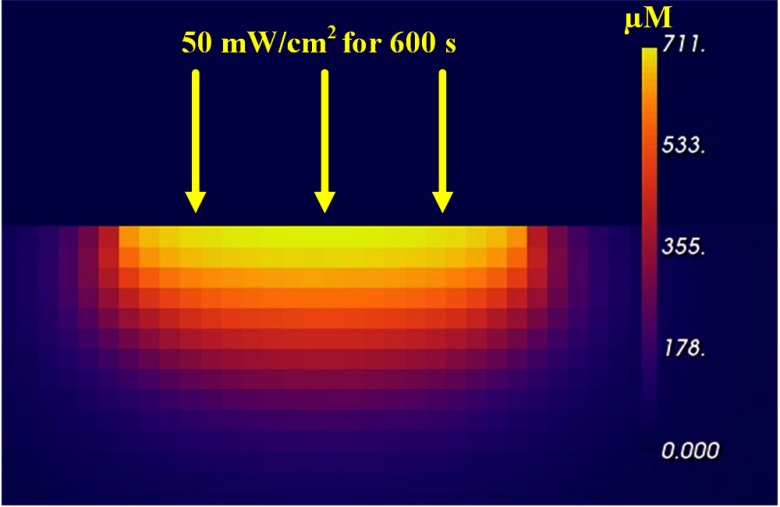
YZ slice of Dosie™’s PK [O12]rx results through the center of the target volume shown for an incident fluence rate of $50\;\mathrm{mW}/{\mathrm{cm}}^{2}$ for 600 s and for 0.50-mm voxels.

After the MC calculation of the fluence rate for each voxel of the target, the PK-module performs PK numerical calculations to find [O12]rx for each such voxel of the target volume for a treatment time of 600 s. The YZ slice at the center of the full 3-D simulation is shown in Fig. 5. The maximum [O12]rx just below the surface at the center of the beam is 711  μM or 0.711 mM. The Dosie™ PK runtime was 12 s using 4 CPU processor cores.

To insure each tumor gets a sufficient dose through the full 3-mm tumor thickness, we determined the singlet oxygen dose at a distance of 3 mm below the surface of the target at the center of the illumination. For PK simulations with a 1-mm grid, one needs to look at the third and fourth layers of 1-mm-thick voxels at the center of illumination and to average the two results to get the singlet oxygen dose 3 mm below the target surface. For a 0.50-mm grid, as shown in Fig. 5, this was done by looking at the sixth and seventh layers and by averaging the two results to get the singlet oxygen dose at the interface 3 mm below the target surface. For a 0.25-mm grid, this was done by averaging the values from the 12th and 13th layers.

The final Penn and Dosie™ simulation results for all the mice groups are shown in Table 2. Columns 8, 9, and 10 include Dosie™ PK calculated values of [O12]rx at 3 mm below for different grid resolutions: 1.0, 0.5, and 0.25 mm voxels, respectively. The numbers in parentheses show the mismatch with the corresponding Penn results (see column 7). Except for mice groups 1 and 2, the differences between the PK results and the Penn results are less than about 2%.

**Fig. 6:**
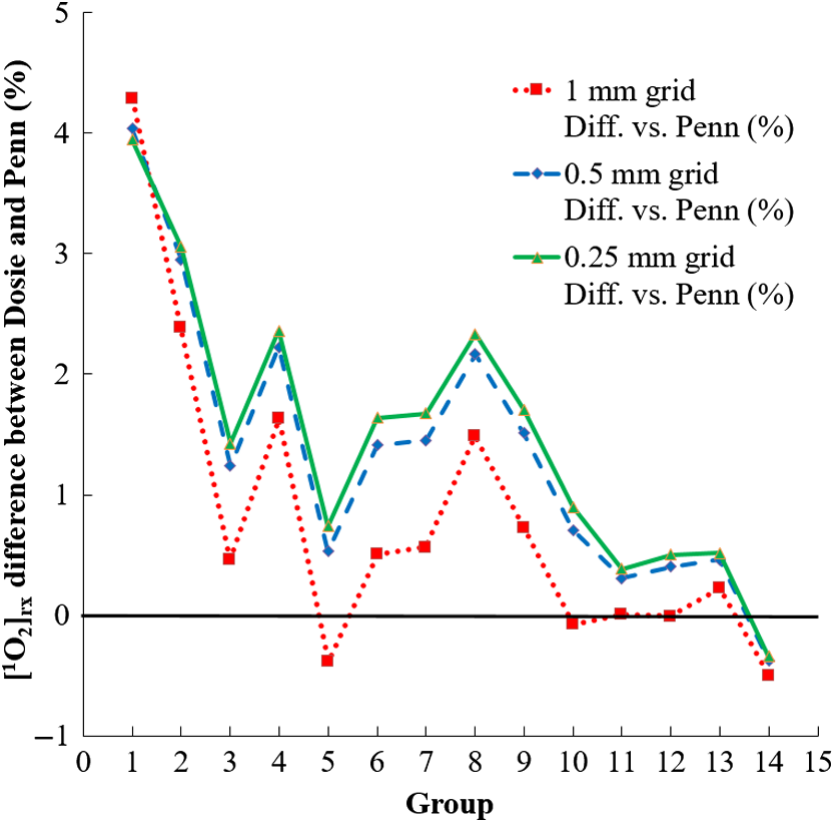
Comparison of Dosie™’s PK results to Penn results for 1-mm, 0.50-mm, and 0.25-mm voxels.

Plots showing the differences between the PK results and the Penn results are shown in Fig. 6. In going from 1.00-mm voxels to 0.50-mm voxels, the differences with the Penn results change on the order of 1%. However, in going from 0.50-mm voxels to 0.25-mm voxels, the differences only change ∼0.2%, indicating that reducing the grid step below 0.50 mm is unnecessary.

## 5 Discussion

**Fig. 7:**
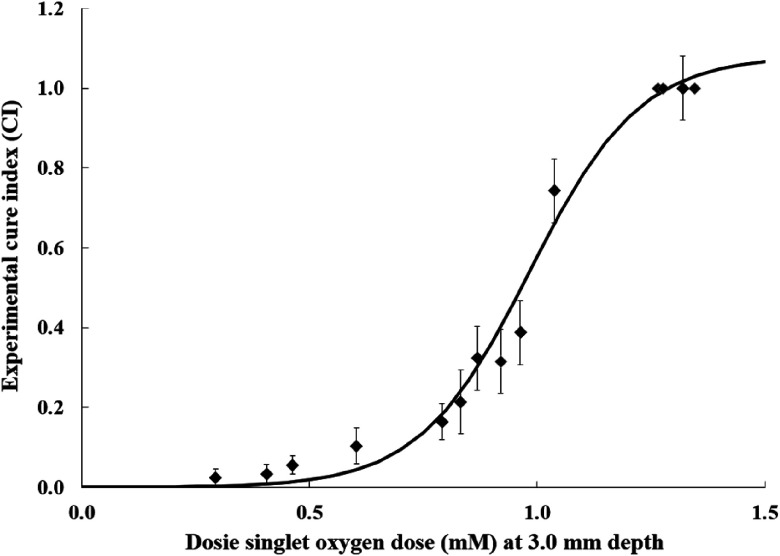
Penn experimental cure index (CI) data from Ref. 10 versus Dosie™ calculated singlet oxygen dose, [O12]rx, at 3 mm below the target surface for 0.50-mm grid. The CI error bars and the expression for the solid line, CI=1.08/(1+3490×exp(−8.301×[O12]rx)) are from Ref. 10.

**Fig. 8:**
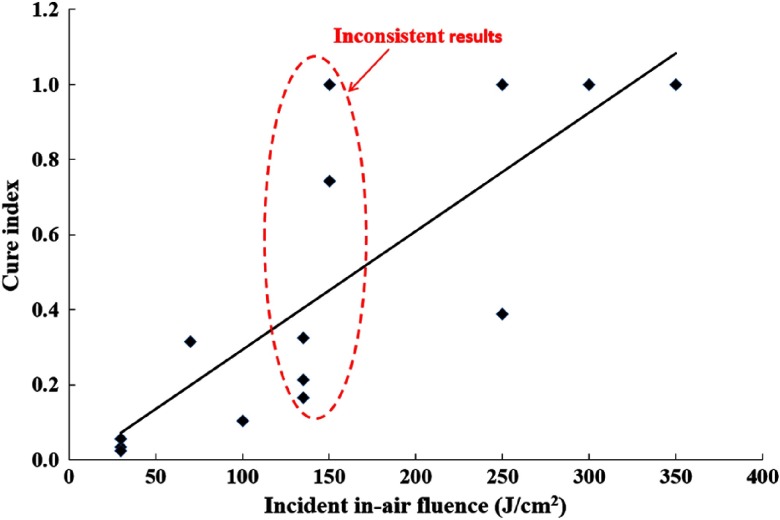
Cure index versus conventional light dose (in-air incident fluence on the target surface). Data taken from Ref. 10.

To determine whether the singlet oxygen dose is a better PDT outcome predictor than the conventional light dose, we plot the cure index versus reacted singlet oxygen values [O12]rx, which are calculated by Dosie™ at 3 mm below the surface (Fig. 7), and versus the incident in-air fluence (Fig. 8). The cure index data are taken from the last column of Table 2.

Figure 7 shows a strong correlation of CI with singlet oxygen dose. Note that CI=1.0 (i.e., cure) indicates no tumor regrowth during the 14 days of the tumor monitoring. Furthermore, Fig. 7 shows that a threshold dose of [O12]rx of ∼1.3  mM is required in order to achieve a CI=1.0. Significantly, lower singlet oxygen doses can allow the tumors to quickly regrow.

Figure 8 shows the poor correlation of CI with incident in-air fluence on the target surface. For example, for a narrow domain of incident fluence values of $135-150\;{J/\mathrm{cm}}^{2}$, the CI values ranged from a poor value of about 0.16 to an excellent value of 1.0, indicating low correlation with fluence. Moreover, a good outcome has been observed for the incident fluence ranging from 150 to $350\;{J/\mathrm{cm}}^{2}$, which makes it impossible to localize a “safe” range to use for the light dose during pretreatment planning. Singlet oxygen dose clearly shows a better correlation.

At this time, we can suggest some possible additional applications of Dosie™. For example, it may be useful for designing and trying-out preclinical studies (i.e., animal trials), thus reducing the time and costs associated with expensive trials that can cost hundreds-of-thousands of dollars and months or years. One could use Dosie™ to try hundreds of possibilities before incurring major costs. Additionally, Dosie™ may be used for certain superficial clinical cancer treatments in research hospitals, for cancers such as esophagus, endobronchial, oral cavity, or skin. Research hospitals are performing clinical studies in esophagus (Mem. Sloan-Kettering, New York, NCT03133650), oral cavity (Roswell Park Cancer Institute, New York, NCT02119728), and certain skin conditions (Cleveland Clinic, Ohio, NCT 02124733).

## 6 Conclusion

PDT treatment results depend on many variables, such as tissue oxygenation, PS concentration, tissue optical parameters, PK parameters, incident fluence rate, and treatment time. A reliable and accurate dosimetry method that takes these variables into account is needed in order to make PDT outcomes predictable. The Dosie™ simulation results in this study are consistent with Penn results^10^ that validate the use of Dosie™ to predict BPD-mediated PDT results on mice animal studies. Furthermore, the results indicate that calculated singlet oxygen dose, [O12]rx, is a very good predictor of PDT outcomes, whereas incident in-air fluence—the conventional light dose—may be a poor predictor. The results indicate that planning and monitoring of PDT treatments should preferably utilize simulations of singlet oxygen dose rather than light dose in order to better predict and improve treatment outcomes.
